# Myths and Facts Regarding Low-Carbohydrate Diets

**DOI:** 10.3390/nu17061047

**Published:** 2025-03-17

**Authors:** Nina Teicholz, Steven M. Croft, Ignacio Cuaranta, Mark Cucuzzella, Mariela Glandt, Dina H. Griauzde, Karen Jerome-Zapadka, Tro Kalayjian, Kendrick Murphy, Mark Nelson, Catherine Shanahan, Jodi L. Nishida, Robert C. Oh, Naomi Parrella, Erin M. Saner, Shebani Sethi, Jeff S. Volek, Micalla Williden, Susan Wolver

**Affiliations:** 1 Independent Researcher, New York, NY 10024, USA; 2 Independent Researcher, Houston, TX 77074, USA; 3 Independent Researcher, Rosario 2000, Argentina; 4 Department of Family Medicine, West Virginia University School of Medicine, Morgantown, WV 26506, USA; 5 Martinsburg Veterans Administration Hospital, Martinsburg, WV 25405, USA; 6 OwnaHealth, New York, NY 10006, USA; 7 Department of Internal Medicine, University of Michigan School of Medicine, Ann Arbor, MI 48109, USA; 8 VA Ann Arbor Healthcare System, Ann Arbor, MI 48105, USA; 9 Valley Gastroenterology Associates, Beaver Falls, PA 15010, USA; 10 Trajectory Health Partners, Mars, PA 16046, USA; 11 Greenwich Hospital, Yale New Haven Health, Greenwich, CT 06830, USA; 12 Western North Carolina VA Health Care System, Asheville, NC 28805, USA; 13 Independent Researcher, Chicago, IL 60174, USA; 14 Rebel Well, LLC, Orlando, FL 32803, USA; 15 The Keto Prescription Clinic, Honolulu, HI 96734, USA; 16 Department of Medicine, Stanford University School of Medicine, Palo Alto, CA 94305, USA; 17 VA Palo Alto Health Care System, Palo Alto, CA 94304, USA; 18 Department of Family and Preventive Medicine, Rush Medical College, Chicago, IL 60612, USA; 19 Department of Surgery, Rush Medical College, Chicago, IL 60612, USA; 20 Department of Family & Community Medicine, Wake Forest University School of Medicine, Winston-Salem, NC 27157, USA; 21 Metabolic Psychiatry, Department of Psychiatry and Behavioral Sciences, Stanford University School of Medicine, Stanford, CA 94305, USA; 22 Department of Human Sciences, The Ohio State University, Columbus, OH 43210, USA; 23 Independent Researcher, Auckland 0618, New Zealand; 24 Department of Internal Medicine, Virginia Commonwealth University School of Medicine, Richmond, VA 23298, USA

**Keywords:** low-carbohydrate diet, ketogenic, diabetes, obesity, heart disease

## Abstract

As the prevalence of chronic diseases persists at epidemic proportions, health practitioners face ongoing challenges in providing effective lifestyle treatments for their patients. Even for those patients on GLP-1 agonists, nutrition counseling remains a crucial strategy for managing these conditions over the long term. This paper aims to address the concerns of patients and practitioners who are interested in a low-carbohydrate or ketogenic diet, but who have concerns about its efficacy, safety, and long-term viability. The authors of this paper are practitioners who have used this approach and researchers engaged in its study. The paper reflects our opinion and is not meant to review low-carbohydrate diets systematically. In addressing common concerns, we hope to show that this approach has been well researched and can no longer be seen as a “fad diet” with adverse health effects such as impaired renal function or increased risk of heart disease. We also address persistent questions about patient adherence, affordability, and environmental sustainability. This paper reflects our perspective as clinicians and researchers engaged in the study and application of low-carbohydrate dietary interventions. While the paper is not a systematic review, all factual claims are substantiated with citations from the peer-reviewed literature and the most rigorous and recent science. To our knowledge, this paper is the first to address potential misconceptions about low-carbohydrate and ketogenic diets comprehensively.

## 1. Introduction

The prevalence of chronic diseases continues to rise, with 93% of American adults having risk factors or taking medication for obesity, diabetes, or heart disease, according to a recent estimate [1]. This public health emergency requires doctors and other experts to remain open-minded about new evidence-based approaches to these stubborn epidemics. Low-carbohydrate diets have been studied for nearly three decades, with papers on clinical trials now numbering in the thousands [2,3]. These trials have demonstrated significant benefits for the prevention and treatment of obesity, diabetes, cardiovascular disease, hypertension, and mental health disorders, among many other chronic diseases [4]. The American Diabetes Association (ADA) [5], Diabetes Canada [6], the European Association for the Study of Diabetes [5], the Australian Diabetes Association [7], and an ADA-supported consensus report [8] now recognize low-carbohydrate eating patterns as being acceptable for managing type 2 diabetes, although generally these groups still find a low-calorie approach preferable. The Obesity Medicine Association noted, in its most recent scientific statement, that “[m]any patients with pre-obesity/obesity who undergo weight reduction via carbohydrate-restricted diets may experience improvement in fat mass, disease symptoms, and/or improvement or remission in diabetes mellitus, hypertension, dyslipidemia (i.e., triglycerides), and thus reduced CVD risk factors” [9]. Further, the American Heart Association (AHA) has stated that a very low-carbohydrate diet “versus moderate carbohydrate diets yield a greater decrease in A1c, more weight loss and use of fewer diabetes medications in individuals with diabetes” [10]. The biological mechanisms for the unique benefits of carbohydrate restriction have been extensively described [11].

Although low-carbohydrate diets have been officially recognized, the current literature often fails to reflect recent scientific findings. For example, the AHA, in discussing the ketogenic diet in a 2023 scientific statement, highlighted the problem of the “keto flu… [which] improve[s] over time”, but the diet was assigned a low ranking, partly because flu-like symptoms were considered to be likely to impair adherence [12]. The paper did not mention that methods for avoiding the keto flu have been published since 2011 [13] and in the peer-reviewed scientific literature since 2018 [14].

Similarly, dozens of epidemiological studies have reported increased mortality linked to low-carbohydrate diets. However, a 2021 analysis of 14 papers [4] found that the diets in these papers were not “low-carbohydrate” according to the definition used by researchers in the field since 2015, which limits carbohydrates to 25–26% of calories [15]. The 14 papers allowed for up to 37%. Interestingly, the world’s largest observational study, which included 135,335 individuals across 18 countries, found that higher carbohydrate intake was associated with an increased risk of total mortality [16].

This paper addresses these and other misunderstandings so that patients and practitioners interested in low-carbohydrate diets can be more informed about this choice. Any nutritional approach should be selected based on up-to-date information and a patient’s desires and preferences. Seeking the guidance of an experienced low-carbohydrate clinician is also recommended. This paper reflects the opinion of its authors and should not be regarded as a systematic review of these topics.

Any discussion of a dietary approach requires using accurate and current definitions. Although different standards exist, leading researchers and practitioners in the field have converged upon a definition of a “low-carbohydrate” diet as one that allows for no more than 130 g of carbohydrate per day, or 25% of calories [4,17] (Table 1). A “ketogenic” or “keto” diet is defined as having 20–50 g of carbohydrates daily, or less than 10% of calories. This paper will use the term “low-carbohydrate diets” to refer to both approaches.

**Figure 1:**
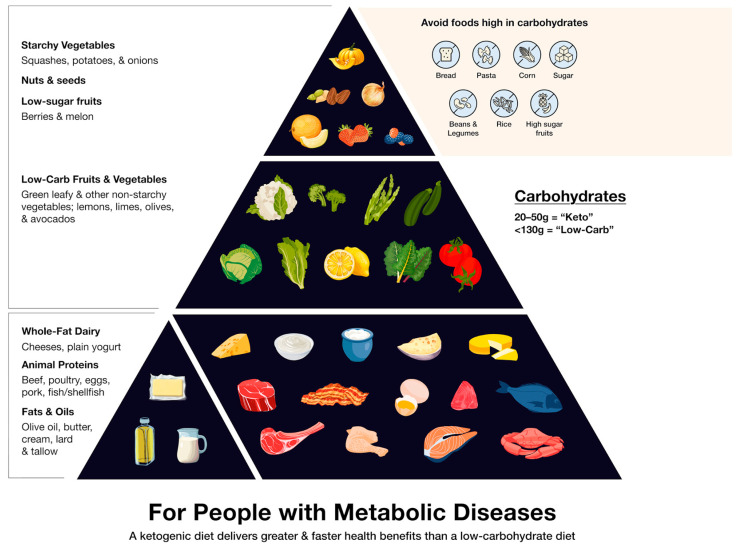
Low-carb/ketogenic food pyramid.

Low-carbohydrate diets can include a wide range of whole foods (Figure 1).

## 2. Materials and Methods

Contributors to this paper are primarily clinicians with a wealth of experience in actively counseling patients on low-carbohydrate diets. The authors identified the concerns listed below as those most often encountered in conversations with patients and other clinicians, which this paper aims to address. Note that this paper mainly avoids using observational or epidemiological studies to substantiate claims, because this type of study, while establishing associations, cannot reliably establish cause and effect relationships. Most of the data cited in the paper are from clinical trials, a far more reliable form of evidence.

## 3. Concerns About Low-Carbohydrate Diets

### 3.1. Side-Effects

As mentioned above, flu-like symptoms, including fatigue, headaches, and muscle aches, have long been a concern for people starting a low-carbohydrate diet. These symptoms are usually due to the excretion of sodium in the urine and the consequent diuretic effect of carbohydrate restriction that manifests when blood volume is decreased (hypovolemia). The condition can easily be alleviated or avoided simply by drinking two cups of soup broth daily (even soup derived from a bouillon cube) or obtaining other sources of sodium and essential minerals.

Ketoacidosis is often raised as another possible side-effect, yet this condition mainly occurs in people with type 1 diabetes when insufficient insulin is present [18]. Rarely, a different condition called euglycaemic ketoacidosis is an adverse effect associated with sodium–glucose co-transporter-2 inhibitors (SGLT2i) in people with diabetes [19]. However, the state of nutritional or physiological ketosis, where ketone bodies are present and the body burns fat for fuel, is a normal state of human physiology and does not cause this condition [20].

### 3.2. The Human Need for Carbohydrates

Many clinicians are concerned that low-carbohydrate diets are not “balanced”. An important concept is that people with metabolic diseases such as obesity and type 2 diabetes cannot consume the same range of foods as those who are healthy, i.e., a person with established type 2 diabetes cannot eat as liberally as a healthy 19-year-old. The concept of personalized nutrition reflects the fact that nutrition must be tailored to individual needs; this includes a person’s degree of metabolic dysfunction. Many studies have established that people with chronic diseases suffer from carbohydrate intolerance. Thus, in the same way that people with gluten intolerance avoid gluten, those with carbohydrate intolerance must limit carbohydrates.

There are no deficiency symptoms that occur even in the complete absence of dietary carbohydrates [21]. The small amount of glucose needed for the functioning of the brain, red blood cells, and the eyes can be created using other substrates via a process called gluconeogenesis [22]. The National Academies of Sciences concluded in a 2005 report that the essential amount of carbohydrate is zero [23].

### 3.3. Heart Disease

The belief that saturated fats increase heart disease risk has been challenged. In the context of lower carbohydrate intake, several studies have shown that increased saturated fat consumption by two- to three-fold either has no effect or decreases the abundance of saturated fatty acids in the blood [24]. Furthermore, a 2020 “State of the Art” review of saturated fat in the authoritative Journal of the American College of Cardiology (JACC) found “no beneficial effects of reducing SFA [saturated fat] intake on cardiovascular disease and total mortality” and little-to-no effect on cardiovascular events [25]. These findings have been confirmed in nearly two dozen systematic reviews and meta-analyses of large clinical trials [26]. For patients who prefer not to eat animal fats, they should know that a low-carbohydrate diet with plant-based fats is possible [27].

A related concern is the rise in LDL-cholesterol (LDL-C) often seen in low-carbohydrate diets. However, a recent meta-analysis of 41 low-carbohydrate diet trials found that mostly lean people (BMI < 25) see this type of cholesterol rise [28]. Even the increase in this group may not signify an increased risk of heart disease, since a recent study in JACC Advances found that these lean outliers with high LDL-C had no significant plaque build-up after 4.7 years compared to a matched control group [29]. This study suggests that elevated LDL cholesterol on low-carb diets is not meaningful for observable heart disease. By contrast, a substantial body of published work over the past 20 years has documented that low-carbohydrate diets induce favorable changes in many cardiovascular risk markers, including high triglycerides, low HDL-cholesterol, increased small, dense LDL particles, high blood sugar, hyperinsulinemia, hypertension, and chronic inflammation, in addition to reducing stroke risk. A large clinical trial on the ketogenic diet for one year found that, of the 20 heart disease risk factors measured, 17 showed significant improvements, while 2 remained unchanged [30]. LDL-C was the sole risk factor that worsened. Overall, the 10-year atherosclerotic cardiovascular disease (ASCVD) risk score for these subjects decreased by 11.9%. In another small low-carbohydrate intervention that did not restrict saturated fat intake, the 10-year cardiovascular risk was reduced by 44% [31]. Altogether, these improvements can be seen as compensating for any potentially concerning rise in LDL-C.

The higher red meat consumption, common to low-carbohydrate diets, is also thought to cause both heart disease and cancer. However, the most rigorous comprehensive reviews of the data on red meat, using a gold-standard methodology called “GRADE” (Grading of Recommendations Assessment, Development and Evaluation), concluded that there is very little high-quality evidence to justify any health concerns about red meat [32,33,34,35]. These reviews found that the available evidence is of “low” to “very low” certainty for health outcomes, including heart disease, type 2 diabetes, and cancer of any kind. In other words, the best available evaluation of the existing evidence does not support concerns that red meat causes these diseases. News headlines and many studies reporting contrary information are based almost exclusively on low-quality evidence from observational or mechanistic studies rather than high-quality evidence from clinical trials on red or processed meat.

### 3.4. Type 2 Diabetes

While clinicians commonly believe type 2 diabetes is an irreversible condition, the ADA has established that remission of this disease is possible [36] and that reducing carbohydrate intake has the “most evidence” for glycemic control [8]. A clinical trial on 238 participants with type 2 diabetes for a mean of 8 years found that more than 50% reversed this disease on a ketogenic diet, with most reducing or eliminating medications in just 10 weeks [37]. These results were sustained for the two-year duration of the trial [38]. An audit of a primary-care practice in England also found more than 50% remission for 186 patients who chose to follow a ketogenic diet [39].

It is important to monitor insulin closely when a patient is reducing carbohydrates. Peer-reviewed guides on deprescription during the use of low-carbohydrate diets are available for practitioners [40,41,42].

The use of medication to treat type 2 diabetes is often thought to be preferable to diet. However, many medications, including insulin, sulfonylureas, and thiazolidinediones often lead to weight gain [43], and disease progression, with very few patients experiencing remission. While GLP-1 agonists may be helpful, these are associated with serious side effects, including gastroparesis and pancreatitis [44], and in some studies have been found to have high discontinuation rates (>50%) [45].

### 3.5. Other Disease Conditions

Various other health conditions have traditionally been thought to worsen while on low-carb diets, such as gut health. However, patients with gastroesophageal reflux disease (GERD) have seen their symptoms improve on a ketogenic diet [46,47]. A pilot study on women with obesity found that higher amounts of carbohydrates worsened GERD, while a high-fat low-carbohydrate diet reduced symptoms [48]. In one study, a zero-fiber diet was found to resolve constipation, compared to higher-fiber diets, which did not [49]. Finally, a recent clinical trial in Sweden published in *The Lancet Gastroenterology and Hepatology* found that a low-carbohydrate diet was just as effective as the well-known “low-FODMAP” approach for reducing symptoms of irritable bowel syndrome (IBS) [50] (FODMAP stands for “fermentable oligosaccharides, disaccharides, monosaccharides and polyols”).

Damage to the kidneys is another concern related to the perception that low-carb diets are higher in protein. However, a correctly formulated low-carb diet is high in fat and moderate in protein. A recent systematic review pointed to several studies showing that ketogenic diets can be therapeutic for kidney disease [51]. The authors also concluded that the diet “can be safely prescribed in patients with type 2 diabetes for treating and remitting diabetes even if they have underlying stage 2 or 3 chronic kidney disease or reduced kidney function”. Even diets higher in protein were not found to damage healthy kidneys in a 2018 meta-analysis [51].

There are questions about the effect on thyroid function and notably lowered plasma T3 (triiodothyronine) found in low-carbohydrate diets. Evidence is limited, since trials have been short-term and limited to specific populations. A cross-over clinical trial found that, despite lowered T3, subjects on a low-carbohydrate diet maintained their metabolic rate and lost more weight than when following a diet high in carbohydrates [52]. A recent systematic review on obesity-related thyroid dysfunction concluded that “the evidence currently supports using [a very low carbohydrate diet] as they can mediate favorable outcomes” [53]. More research is needed.

With regard to gallstones, multiple clinical trials have found that diets higher in fat prevent gallstone formation [54,55]. By contrast, diets low in fat increase gallbladder volume and may increase the risk of gallstone development [56].

Finally, the low-carbohydrate diet is thought by some experts to reduce lifespan (increase mortality). However, the observational studies reporting higher mortality rates on low-carb diets incorrectly define the diet as having 37% of calories or more as carbohydrates, as discussed above, which is not a “low-carbohydrate” diet. These studies are also a weak form of evidence that can only rarely establish cause-and-effect relationships, and they are contradicted by mouse experiments using a ketogenic diet, which found reduced mid-life mortality [57] and increased total lifespan compared to controls [58].

### 3.6. Other Dietary Approaches

Although vegan diets are often considered to be the best for disease reversal, there are surprisingly few clinical trials on this approach, and existing trials tend to be flawed. For instance, the renowned Ornish et al. study, which reported that a vegan diet reversed heart disease, was confounded by interventions other than diet, including exercise, stress management training, smoking cessation, and vitamin supplements [59], while the controls were provided with none of these. Systematic reviews and meta-analyses have found that plant-based diets often lower HDL-C [60,61,62], or have no effect [61], which implies increased cardiovascular risk. The vast majority of evidence used to support vegan diets comes from observational studies, which, as explained, yield low-quality data. Since vegans are frequently health-conscious people who tend to smoke less, consume less alcohol, exercise more, and be of higher socio-economic status [63], it is difficult for studies to isolate the effect of diet alone.

The medical establishment has also had a longtime preference for a low-fat diet, based on the 1970s’ hypothesis that this way of eating could prevent obesity since fat has nine calories/gram versus the four calories/gram in protein or carbohydrate [64]. However, multiple large long-term controlled experiments could not confirm that the low-fat diet led to significant weight loss [65,66]. In head-to-head trials with low-carbohydrate diets, the latter have nearly always led to more weight loss than those low in fat [67,68,69]. Further, the U.S. Dietary Guidelines for Americans have not included “low-fat” in their dietary recommendations since 2015 [70].

### 3.7. Sustainability, Cost, and Nutritional Adequacy

While many health practitioners believe that low-carbohydrate diets are unsustainable, a 2017 survey of 1580 people found that a majority had sustained a low-carb diet, defined as <100 g of carbohydrates per day, for more than a year, and 34% reported sustaining the diet for more than two years [71]. Furthermore, those on low-carbohydrate diets for two years or more said that they had largely maintained their weight loss. The diet is sustainable because protein and fat are highly satiating, allowing for patients to be hunger-free between meals. Respondents reported being highly motivated to stay on the diet due to visible health improvements.

The view that low-carbohydrate diets are excessively expensive is challenged by a 2019 cost analysis comparing a low-carbohydrate diet with the New Zealand government’s recommended guidelines (which are almost identical to those in the US [72]), which found that the former cost only an extra USD 1.27 per person per day [73]. Ground beef and eggs are examples of inexpensive sources of food on this diet. Furthermore, a recent Medscape article entitled “For Richer, For Poorer: Low-carb Diets Work For All Incomes” described a pilot trial of 100 low-income participants in the South Bronx, the poorest borough in New York City, including one woman in a homeless shelter, who successfully adopted this diet [74]. A free book, *Low-carb For Any Budget*, is available for download [75].

The idea that low-carbohydrate diets are nutritionally deficient is contradicted by studies finding this nutritional approach to be replete in all the essential minerals and vitamins [76,77], including for children [78]. Any concern about the lack of vitamin C can be allayed by consuming low-carbohydrate vitamin-C-rich fruits, such as lemons, limes, and tomatoes. Because glucose interferes with vitamin C absorption [79], a low-carbohydrate diet, which is low in glucose, is thought to reduce the need for this vitamin. By contrast, the dietary patterns recommended by U.S. Dietary Guidelines for Americans “do not meet Recommended Dietary Allowance or Adequate Intake goals [for] the following: Iron, Vitamin D, Vitamin E, Choline, and Folate”, according to the government’s expert report on the guidelines report from government experts [80].

### 3.8. Other Concerns

Climate concerns are a frequent objection to higher beef consumption. Low-carbohydrate diets need not be high in red meat.. Beyond that, scientists debate the implications for climate resilience and eco-system protection,, with many soil scientists regarding livestock as essential to the environment, especially when raised using regenerative practices [81]. A 2019 report by the U.S. Environmental Protection Agency calculated that livestock generate just 3.9% of total U.S. greenhouse gas emissions [82]. Even accepting the oft-cited far-higher numbers for livestock emissions, patients should be given a choice about whether to eat meat or save on emissions in other ways, such as driving fewer miles or reducing plane travel.

Finally, low-carbohydrate diets have not been shown to be deleterious to athletes. Studies show that the ketogenic diet has helped athletes improve their body composition, trim fat, maintain performance, and improve recovery. These studies have included marathon runners [83], CrossFit athletes [84], elite gymnasts [85], and others performing high-intensity exercise [86] or interval training [87], as well as military personnel [88].

## 4. Discussion

This paper seeks to address the major concerns about low-carbohydrate diets. Given the ongoing crisis of obesity and diabetes, among other chronic diseases, health practitioners should be up-to-date on evidence-based methods for combating these diseases. This paper demonstrates that common concerns about low-carbohydrate diets being unsafe, unhealthy, or unsustainable are not supported by the most rigorous scientific literature. Evidence-based diets, including those low in carbohydrates, should be supported for patients who choose them.

## 5. Conclusions

- The low-carbohydrate (or ketogenic) diet is supported by a large body of clinical trial research demonstrating its safety and efficacy.
- Commonly held concerns, such as the idea that low-carbohydrate diets increase mortality or increases the risk of heart disease, are not supported by the evidence.
- There are no harmful side effects of low-carbohydrate diets.
- The “keto flu” that some patients experience at the start of the diet can be treated and avoided.
- Low-carbohydrate diets can be sustainable and nutritionally complete.

## Figures and Tables

**Table 1:** Definitions of low-carbohydrate diets.

Diet	Carbohydrates as % of Daily Calories	Grams of Carbohydrates Daily
Low-carbohydrate	25%	130 or less
Ketogenic (“keto”)	10%	20–50
